# A Randomized Controlled Trial Examining the Effect of Topical Refrigerant Spray on the Perception of an Instantaneous Noxious Stimulus

**DOI:** 10.1016/j.jhsg.2023.03.006

**Published:** 2023-04-14

**Authors:** Brian Gu, Ryan Williams, Jake Rodgers, Blair Boehnke, Jeffrey Rodgers

**Affiliations:** ∗ DMOS Orthopaedic Centers, West Des Moines, IA; † DMU College of Osteopathic Medicine, Des Moines, IA

**Keywords:** Injections, Noxious stimulus, Pain experience, Topical anesthetic, Topical refrigerant

## Abstract

**Purpose:**

Topical refrigerant spray is commonly used with routine hand injections despite mixed evidence about its efficacy in reducing the pain experience. We hypothesize that the use of topical refrigerant spray decreases the pain experience of an instantaneous noxious stimulus.

**Methods:**

Eighty adult volunteer participants were enrolled in the study. We constructed an instrument using the smooth end of a Kirschner wire mounted to the spring of a ballpoint pen to apply an instantaneous noxious stimulus to the long (middle) finger proximal nail fold. Participants completed two trials and were randomized to receive the topical refrigerant spray before either the first or second stimulus and on either the left or right side. Participants were asked to rate the pain of each experience using an 11-point Likert scale and indicate which condition they preferred, if any.

**Results:**

The mean pain ratings for the spray and no-spray conditions were 2.0 and 4.3, respectively, giving a mean difference of −2.3 (*P* < .001, α = 0.05). Subgroup analysis showed no significant effect of sex or medical versus nonmedical occupation (*P* = .28 and .11 respectively) on the mean difference in pain rating between the two conditions. Participants who received the spray first had a higher mean difference in pain rating (2.7) than that in those who received it second (1.9). Fifty-nine participants preferred the spray, whereas 21 participants either preferred no spray or had no preference (*P* < .0001).

**Conclusions:**

The use of topical refrigerant spray significantly decreased the perception of pain from an instantaneous noxious stimulus. A significant majority of participants also preferred the topical refrigerant spray condition. The use of topical refrigerant spray for painful procedures, such as needle insertions, may improve the overall patient experience.

**Type of study/level of evidence:**

Therapeutic I.

Hand surgeons commonly use topical refrigerant spray before routine hand injections with the belief that it mitigates the pain experienced by patients.^1^ However, the existing literature showed mixed evidence of its effectiveness. When examining pain experiences during venipuncture of the forearm for radial artery cannulation, Rüsch et al^2^ (2017) found that topical refrigerant spray produced significantly lower mean pain scores (*P* = .032) than those produced by lidocaine cannulation. Zeiderman et al^3^ (2018) also tested topical refrigerant spray for use with facial neurotoxin injections and found a statistically significant decrease in pain perception with the spray. Finally, in a meta-analysis of the literature examining the use of topical refrigerant spray during intravenous cannulation, Griffith et al^4^ (2016) determined that the spray reduced pain by a mean of −12.5 mm on a 100-point visual analog scale. In contrast, Franko and Stern^1^ (2017) concluded that the use of ethyl chloride spray was not effective in reducing patient pain during hand injections. Furthermore, they found that the use of ethyl chloride spray may potentially increase pain perception in high-pain anticipators when compared with injections without the spray.^1^

The majority of previous studies examining topical refrigerant spray used a between-subjects design. To attenuate the subjective nature of pain and its variability across individuals, we used a within-subjects design that we believe more reliably assesses the highly subjective nature of pain reporting. Although previous studies^1^ asked their participants to recall the pain of the needle insertion 1 minute after the noxious stimulus, we assessed the pain experience immediately after administration.

Although other studies have examined the pain experience of an entire needle insertion procedure, we used a nonclinical model to simulate the instantaneous pain experience of a needle insertion and eliminate the confounding stimuli of a subsequent medical procedure, for example, the injection of fluid into the tissue. Furthermore, studies using clinical procedures enroll patients with potentially confounding preexisting painful conditions. We enrolled healthy volunteers to experience an isolated noxious stimulus to control for these sources of potential bias. Because pain is not the only factor that contributes to the overall patient experience, we separately assessed the overall favorability of the spray experience independent of pain ratings. In accordance with our clinical observations, we hypothesized that the use of topical refrigerant spray decreases the perception of pain during the application of an instantaneous noxious stimulus.

## Materials and Methods

**Figure 1:**
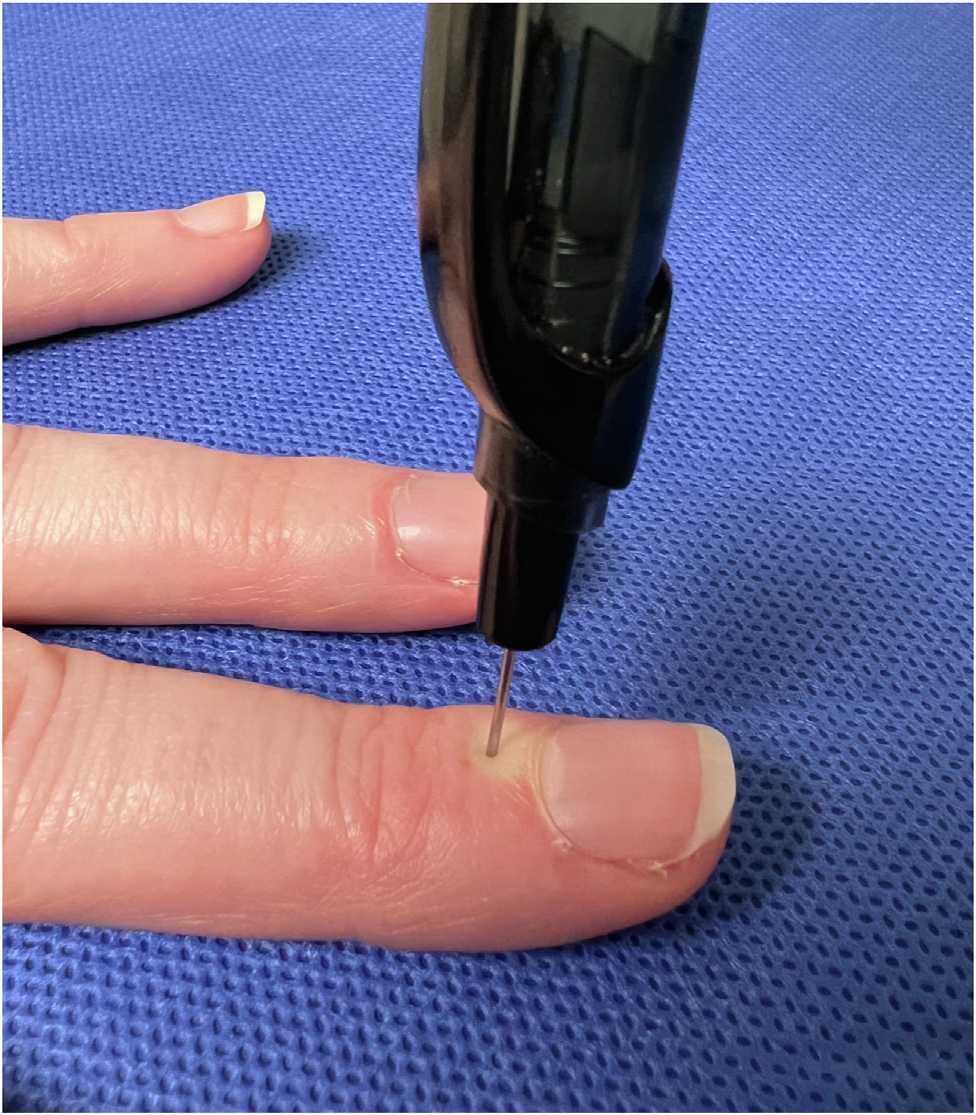
Demonstration of administration of the noxious stimulus device to the proximal nail fold of the middle finger.

We recruited adult (aged ≥18 years) volunteers from September 2021 to November 2021 to participate in an Des Moines University institutional review board–approved study (registration number: IRB-2021-23) in which each participant received an instantaneous noxious stimulus with the use of topical refrigerant spray and another stimulus without the use of spray. We used the Consolidated Standards of Reporting Trials checklist when writing our report.^5^ To achieve at least 80% power and a significance level α of 0.05, we recruited until we reached a sample size of 80 participants. The participants were employees of an orthopedic surgery practice or surgery center and students of a local medical school. Data were collected on each participant’s age, sex, and occupation. Participants were excluded if they had experienced topical refrigerant spray previously for an injection or had a history of peripheral neuropathy. To simulate the experience of a needle insertion, we constructed an instrument using the smooth end of a 0.8-mm Kirschner wire mounted to the spring of a ballpoint pen to apply an instantaneous noxious stimulus to the middle finger proximal nail fold (Fig. 1). The instruments were tested to confirm that they produced 8.9 × 10^6^ Pa of force when the spring of the pen was clicked. Pre-experimental testing of the device confirmed that this was a level of force that consistently produced a sufficient pain stimulus. The participants received the McKesson Topical Anesthetic Spray (1,1,1,3,3-pentafluropropane and 1,1,1,2-tetrafluoroethane). To ensure equal numbers in each testing condition, participants were block randomized to one of four testing sequences and received the stimulus once on each middle finger as follows: (1) spray on the right side, then no spray on the left side; (2) spray on the left side, then no spray on the right side; (3) no spray on the left side, then spray on the right side; and (4) no spray on the right side, then spray on the left side. Participants served as their own controls, with no-spray as the control condition. The spray was administered for 5 seconds on the proximal nail fold. There was no delay between the participant recording their first pain rating and the second stimulus. During the trial, participants were shielded from the application of the topical refrigerant spray and stimulus using a small curtain as a barrier between their vision and hands. Participants were asked to rate the pain of each condition using an 11-point Likert scale (rating from 0 to 10, with 0 being the least and 10 being the most amount of pain) and to indicate which condition they preferred, if any. The responses were collected in a blinded fashion. We had first-aid materials available in the event of unintentional injury or piercing of the skin by the device, but no such harms occurred.

We analyzed the effect of topical refrigerant spray on pain perception by comparing the difference between mean pain ratings in the spray condition and those in the no-spray condition. We also performed a chi-square goodness-of-fit test in which the number of participants who preferred no spray or had no preference was compared with the number of participants who preferred the spray.

We stratified the data by sex, occupation, age, preference, and order of conditions (spray first vs second). For occupational data specifically, we classified physician assistants, orthopedic and radiologic technicians, medical students, nursing professions, and physical therapists as medical occupations. Patient navigators, administrative positions, and other miscellaneous positions were classified as nonmedical occupations. For age stratification, participants were divided into two groups above and below the median age of 32 years. For all the stratified groups, we calculated the difference for each individual between their spray rating and no-spray rating. We then calculated the mean difference for the entire stratified group and analyzed the mean differences for statistical significance. The mean differences were calculated as negative values to maintain consistency in the calculations across the groups.

To test the normality of each subset of data, we used the Kolmogorov–Smirnov test for groups with >30 entries and the Shapiro–Wilk test for groups with <30 entries. If at least one group did not have a normal distribution in the subset of data, we used the Mann-Whitney U test to analyze for statistical significance. For the overall comparison of pain ratings with and without the use of topical refrigerant spray, we used the Wilcoxon signed-rank test. We analyzed the age data according to two groups: one with all participants below the median age of 32 years and the other with all participants above the median age. The *P* value threshold for statistical significance in all tests was .05. Ninety-five percent CIs were constructed for the difference in mean differences for each of the stratified groups.

## Results

Table 1 contains the basic demographic information of the study. Overall, we found a statistically significant mean difference of −2.3 (*P* < .001) in the pain score when comparing no spray with spray for all participants. Other statistically significant results included a mean difference of −0.9 for participants who preferred the no-spray experience (*P* = .058) and a mean difference of −1 when comparing old participants with young ones (*P* = .001). Additionally, when comparing the mean differences in pain score between those who received the spray first versus those who received it second, we found values of 2.7 for first and 1.9 for second. The difference in mean differences, −0.8, was significant (*P* = .021), indicating a greater mean pain difference for those who received the spray first than for those who received the spray second.

**Table 1:** Demographic Information and Basic Results of the Study Population

Characteristic	n	%	Mean	SD (sample)
Total volunteers	80	100	-	-
Sex				
Male	24	30		
Female	56	70		
Age (y)	-	-	35.8	11.7
Occupation				
Health care	61	76.2	-	-
Non–health care	19	23.8	-	-
Occupation (health care)				
Physician assistant	9	14.7	-	-
Orthopedic technician	9	14.7	-	-
Radiology technician	12	19.7	-	-
Medical student	7	11.5	-	-
Physical therapist	7	11.5	-	-
Other provider	17	27.9	-	-
Occupation (non–health care)				
Patient navigator	5	26.3	-	-
Administration	14	73.7	-	-
Mean pain score with no spray	-	-	4.3	2.0
Mean pain score with spray	-	-	2.0	1.4
Preference for spray				
Preferred spray	59	73.8	-	-
Preferred no spray	14	17.5	-	-
No preference	7	8.7	-	-

When examining the preference data, 59 participants preferred the spray, 14 participants preferred no spray, and seven participants had no preference. The chi-square test was statistically significant (*P* < .0001), indicating a preference for the spray.

**Figure 2:**
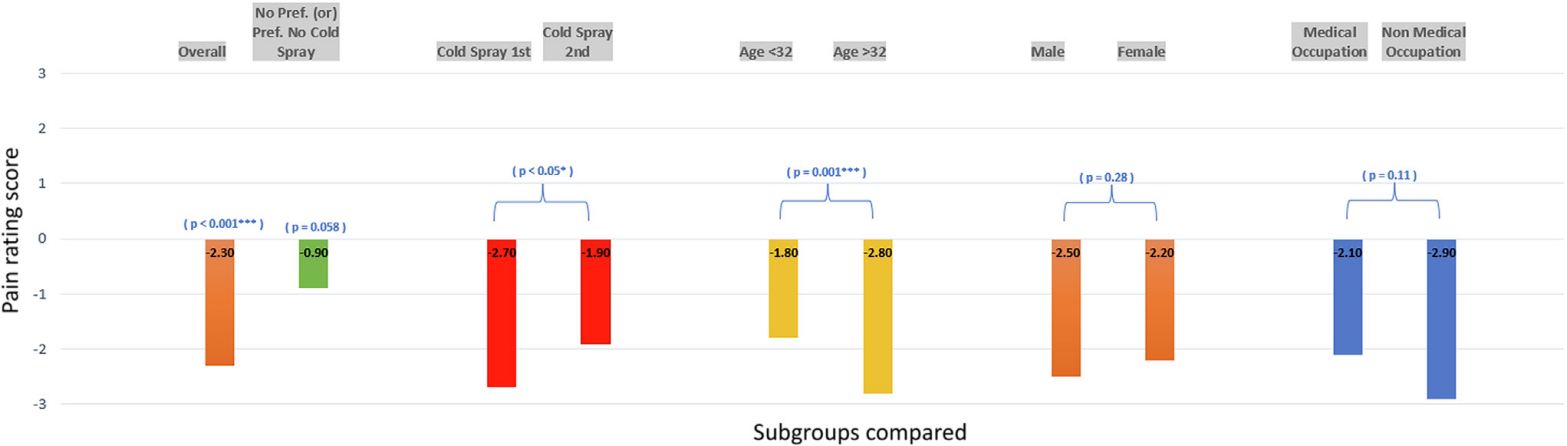
Mean pain rating difference with cold spray.

Please refer to Table 2 and Figure 2 for full results.

**Table 2:** Mean Differences Between Groups and Significance

Characteristic	Mean Difference	*P* value	95% CI
Overall difference	−2.3	5.4 × 10^−13^	−2.9 to −1.8
Overall difference, prefer no spray	−0.9	.058	−1.7 to 0.02
Sex (male vs female)	−0.3	.28	−1.2 to 0.5
Occupation (health care vs non–health care)	−0.8	.11	−1.6 to 0.1
Age (<32 vs >32 y)	−1	.0010	−1.7 to −0.3
Spray first vs spray second	−0.8	.021	−1.5 to −0.08

## Discussion

The goal of our study was to provide clarity on whether topical refrigerant spray is effective in mitigating the pain perceived in response to an instantaneous noxious stimulus. Our nonmedical model of pain maximizes the ability to accurately answer this question. We also assessed the participants’ preference for the spray independent of pain ratings.

Our results demonstrated that topical refrigerant spray was successful in decreasing the perception of pain in response to an instantaneous noxious stimulus and that most participants preferred the overall experience of the spray condition as opposed to the no-spray condition. A subset of participants preferred no spray and had a nonsignificant decrease in the pain experience with the spray (*P* = .058). In the clinical setting, it is important to consult patients on whether they have a preference for treatments, as there are factors such as psychological bias and patient preferences that may contribute to the overall patient experience in addition to pain perception.

There was no statistically significant difference in the mean difference in pain ratings for either subgroup of sex or occupation (*P* = .28 and .11 respectively). Age, however, was a significant factor with older participants demonstrating a greater response to the spray.

It was interesting to note the difference in mean differences when spray was applied in the first trial versus the second trial. Those who received the spray first perceived a greater difference in pain between the spray and no-spray conditions than that perceived by those who received the spray second. The nocebo effect suggests that negative expectations can affect the neuromodulation of pain pathways in such a manner that results in a magnified pain experience,^6^ which may explain this observation. By receiving the perceived therapeutic treatment first, participants may have experienced a heightened anticipation of pain during the second trial.

We noted that some participants preferred the experience without spray even if they rated it as more painful. This may be due to the prolonged duration of the spray stimulus versus the instantaneous nature of the device; some participants may have found a prolonged stimulus less preferable even to a noxious stimulus that was shorter. Another possibility may be the difference in sensation between a temperature-based stimulus and a nociceptive-based stimulus. These participants may have had a higher tolerance for a purely nociceptive stimulus rather than a cold stimulus that may have then triggered a nociceptive response.

There were several limitations to our study design. Although our results showed that there was no significant difference between the pain experiences of medical and nonmedical occupations, it is possible that our study population may have influenced the results. Because we recruited participants from orthopedic clinics, a surgery center, and a medical school, they may have had more prior knowledge and firsthand observations about the spray in clinical practice. Another limitation was the variation in stimulus application. Although the instruments we used were all calibrated to produce a consistent force and we had specific guidelines for the procedure, we had multiple investigators apply the stimulus to participants. It is possible that the experimenters could have varied the speed with which the instrument was clicked or the time interval between stimulus applications.

By controlling for confounding variables in previous studies, we designed a novel model that demonstrated the effectiveness of topical refrigerant spray in reducing the perception of pain from an instantaneous noxious stimulus. Our results contrast Franko and Stern^1^ as they found no difference in their outcome measures between the spray and nonspray groups. By conducting our study in a nonclinical setting and recruiting volunteers, we also believe that we more effectively controlled for anxiety and other emotional aspects that may accompany patients’ clinical visits.

Although we found a similar result for topical refrigerant spray as Rüsch et al^2^ comparing the spray with lidocaine injections, they evaluated pain on the basis of the entire experience, including the actual cannulation of the radial artery, instead of the noxious stimulus of the injection alone. Our study design was most similar to that of Zeiderman et al^3^ as they also compared pain ratings for Botox injections with and without the spray. However, our study may be more directly applicable to hand surgery and conditions of the hand, whereas the study by Zeiderman et al^3^ was based on facial injections.

Our mean pain scores were higher in the control condition than those in the other three studies (4.33 vs 3.08, 4.5, and 3.07 in the studies by Franko and Stern,^1^ Rüsch et al,^2^ and Zeiderman et al,^3^ respectively). This suggests that our device did produce a stimulus comparable with a clinical needle insertion. The mean differences in the comparison studies were −0.2, 1.1, and 1.9, respectively, showing a wide range in pain score reduction likely attributable to differences in study design, such as what purpose the spray was used for.^1^, ^2^, ^3^

Additionally, we found that participants generally preferred the experience of the topical refrigerant spray as opposed to no spray. The similarity in the pain ratings between our study and other needle insertion studies demonstrates the reliability of our nonmedical model in simulating clinical needle insertions.^4^ Randall et al^7^ (2022) proposed minimal clinically important difference values between 1.6 and 1.9 when translating values from the visual analog scale in patients with pain in their hand and upper extremity, suggesting that our difference of −2.3 holds clinical importance. Therefore, we believe that our results are translatable to clinical settings and support the clinical use of topical refrigerant spray in reducing needle insertion pain.
